# Supplementary material for: Seedless fruit in Annona squamosa L. is monogenic and conferred by INO locus deletion in multiple accessions
Source: Plant Reprod. 2023 May 9;37(2):71–84. doi: 10.1007/s00497-023-00464-9 (PMC11180160; doi:10.1007/s00497-023-00464-9)

**Seedless fruit in *Annona squamosa* L. is monogenic and conferred by *INO* locus deletion in multiple accessions**

Plant Reproduction

Bruno Rafael Alves Rodrigues¹, Charles S. Gasser^2^, Samy Pimenta¹, Marlon Cristian Toledo Pereira¹ and Silvia Nietsche^3^*

¹ State University of Montes Claros, Department of Agricultural Sciences, CEP 39401-369, Janaúba, MG, Brazil

² Dept. of Molecular and Cellular Biology, University of California, Davis, Davis, CA 95616 USA

^3^ Federal University of Minas Gerais, Institute of Agricultural Science, CEP 39404-547, Montes Claros, Minas Gerais, Brazil

*Author for correspondence: [silvia.nietsche@gmail.com](mailto:silvia.nietsche@gmail.com)

**Supplemental figures 1-3**

**Supplemental Figure S1.** Determination of ovule phenotype on freshly dissected flowers. (A) Diagrams of *A. squamosa* ovule structure with sections of wild-type (left) and ino mutant (right) ovules redrawn based on images in Lora et al. (2011). (B to E) Images of freshly dissected *A. squamosa* ovules photographed with a stereomicroscope (Leica, M205C) using the Leica Application Suite software (LAS v4.11). (B and C) M_2_ wild type. (D and E) mutant Brazilian seedless. The domed shape of the wild-type and the more pointed-erect shape of the *ino* mutant are easily differentiated. e, embryo sac; f, funiculus; ii, inner integument; n, nucellus; oi, outer integument.


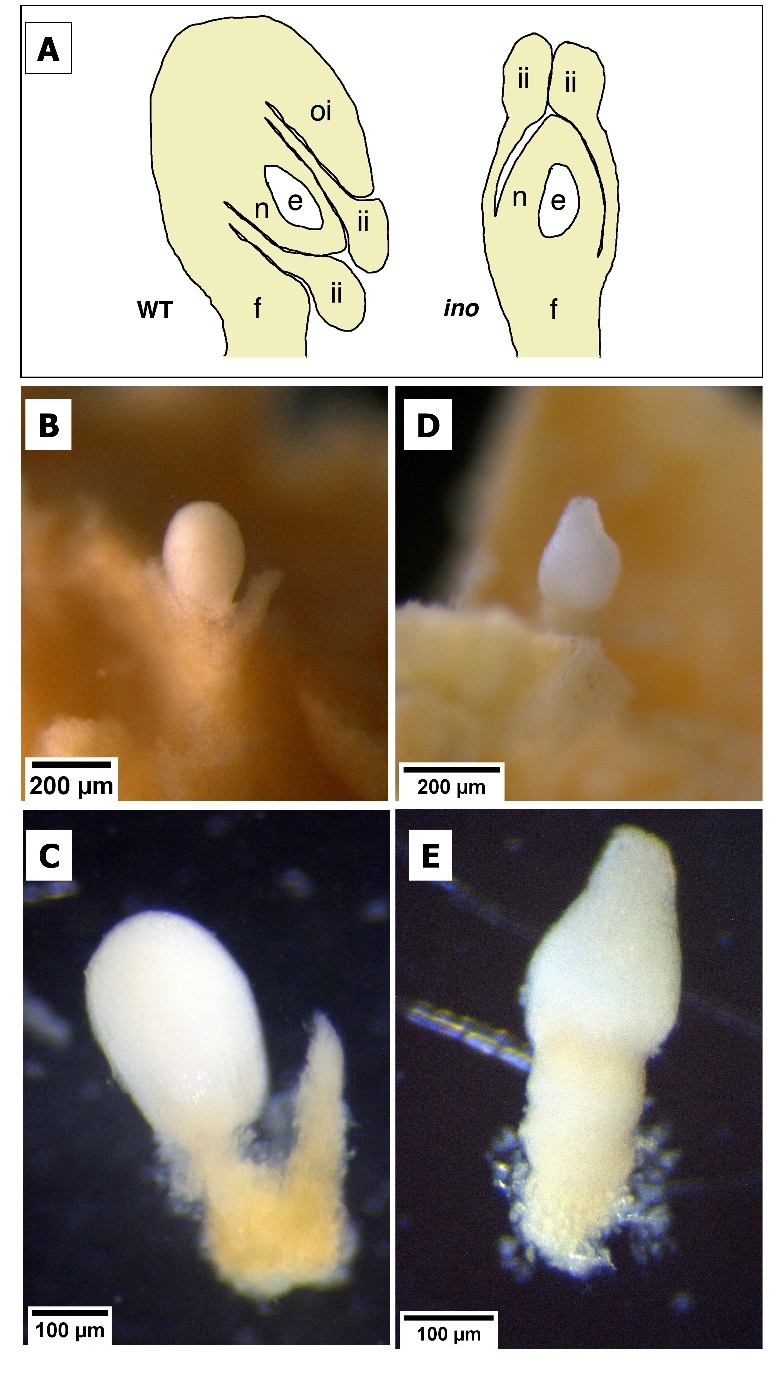


**Supplemental Figure S2.** Alignment produced by BLAST search of whole genome assembly with the previously determined *A. squamosa INO* gene sequence (GenBank GU828033.1).

“INO Gb” is the sequence from GenBank with noncoding flanking sequences and introns in lower case and coding regions in upper case. “Contig” is sequence from the single 587 kb contig (#00001115) identified by BLAST as containing the aligning sequence. The Contig sequence differs from the GenBank entry only in the presence of a 40 bp insertion in the fourth intron, and two single base insertions in the last dozen residues of the alignment over the entire 1847 bp aligned sequence.

INO_Gb 1 atggacaagaacaaagagccataaatcactttctttttcagtagatgaacagatcagaga 60

||||||||||||||||||||||||||||||||||||||||||||||||||||||||||||

Contig 56396 ATGGACAAGAACAAAGAGCCATAAATCACTTTCTTTTTCAGTAGATGAACAGATCAGAGA 56337

INO Gb 61 gagagagagagagagagaggctgttcccgtgttggaaacagaatgagaggaagttactga 120

||||||||||||||||||||||||||||||||||||||||||||||||||||||||||||

Contig 56336 GAGAGAGAGAGAGAGAGAGGCTGTTCCCGTGTTGGAAACAGAATGAGAGGAAGTTACTGA 56277

INO Gb 121 ggagaaaaaaacaactgataagtgcagtctatcactaactctagtcacttcccatgaaaa 180

||||||||||||||||||||||||||||||||||||||||||||||||||||||||||||

Contig 56276 GGAGAAAAAAACAACTGATAAGTGCAGTCTATCACTAACTCTAGTCACTTCCCATGAAAA 56217

INO Gb 181 tctacctcctcaccctagtgaggagaaacctacatctggaccccaattatcttcggccaa 240

||||||||||||||||||||||||||||||||||||||||||||||||||||||||||||

Contig 56216 TCTACCTCCTCACCCTAGTGAGGAGAAACCTACATCTGGACCCCAATTATCTTCGGCCAA 56157

INO Gb 241 ggagcaaatgaaggtgggcctgaagtgatgaatgacccgaatctcacgtataaatgtcat 300

||||||||||||||||||||||||||||||||||||||||||||||||||||||||||||

Contig 56156 GGAGCAAATGAAGGTGGGCCTGAAGTGATGAATGACCCGAATCTCACGTATAAATGTCAT 56097

INO Gb 301 tgttgtagggtttcaatcatgtaaatgaaataaattttctgtatgttaccacccttataa 360

||||||||||||||||||||||||||||||||||||||||||||||||||||||||||||

Contig 56096 TGTTGTAGGGTTTCAATCATGTAAATGAAATAAATTTTCTGTATGTTACCACCCTTATAA 56037

INO Gb 361 gtatcccttgccacacccatcgaacagccacaaagcggttgctttcttttccttctactc 420

||||||||||||||||||||||||||||||||||||||||||||||||||||||||||||

Contig 56036 GTATCCCTTGCCACACCCATCGAACAGCCACAAAGCGGTTGCTTTCTTTTCCTTCTACTC 55977

INO Gb 421 atctgctttcctgttcttcttctttctactATGGACATGTCTACATACAACCACTTCCTT 480

||||||||||||||||||||||||||||||||||||||||||||||||||||||||||||

Contig 55976 ATCTGCTTTCCTGTTCTTCTTCTTTCTACTATGGACATGTCTACATACAACCACTTCCTT 55917

INO Gb 481 GAACTCTCCGACCAGCTTTGCTATGTTCGATGCAGTCATTGCACCACCATTCTTCTAgta 540

||||||||||||||||||||||||||||||||||||||||||||||||||||||||||||

Contig 55916 GAACTCTCCGACCAGCTTTGCTATGTTCGATGCAGTCATTGCACCACCATTCTTCTAGTA 55857

INO Gb 541 ggtgttgttttcggtcatctaatccacgtaccacttacatgactgtagctatcttctgct 600

||||||||||||||||||||||||||||||||||||||||||||||||||||||||||||

Contig 55856 GGTGTTGTTTTCGGTCATCTAATCCACGTACCACTTACATGACTGTAGCTATCTTCTGCT 55797

INO Gb 601 actacagctaatcattcggctactgtgcctctccttccttttattttcattttagGTGAG 660

||||||||||||||||||||||||||||||||||||||||||||||||||||||||||||

Contig 55796 ACTACAGCTAATCATTCGGCTACTGTGCCTCTCCTTCCTTTTATTTTCATTTTAGGTGAG 55737

INO Gb 661 CGTCCCATGCAGCAGCTTGTTGAAGATGGTCACGGTTCGATGTGGCCATTGCACTGGTCT 720

||||||||||||||||||||||||||||||||||||||||||||||||||||||||||||

Contig 55736 CGTCCCATGCAGCAGCTTGTTGAAGATGGTCACGGTTCGATGTGGCCATTGCACTGGTCT 55677

INO Gb 721 CCTTTCTGTTAATGTAATGAAGGCTTCATTTGTTCCACTCCAGCTCCTCGCTTCACTCAA 780

||||||||||||||||||||||||||||||||||||||||||||||||||||||||||||

Contig 55676 CCTTTCTGTTAATGTAATGAAGGCTTCATTTGTTCCACTCCAGCTCCTCGCTTCACTCAA 55617

INO Gb 781 TGATGACCAGgtaatgctggttccaatagcaagtcctatatagctactcttactcaatac 840

||||||||||||||||||||||||||||||||||||||||||||||||||||||||||||

Contig 55616 TGATGACCAGGTAATGCTGGTTCCAATAGCAAGTCCTATATAGCTACTCTTACTCAATAC 55557

INO Gb 841 atcatatgttgaacaaggccagttacattaatcaacgtctttagaaattttctagttaat 900

||||||||||||||||||||||||||||||||||||||||||||||||||||||||||||

Contig 55556 ATCATATGTTGAACAAGGCCAGTTACATTAATCAACGTCTTTAGAAATTTTCTAGTTAAT 55497

INO Gb 901 gcatatgttggtcaatgcagCAAAAGCAAGATCCGTTTGCAGCCTCCCCCATGAAAAATG 960

||||||||||||||||||||||||||||||||||||||||||||||||||||||||||||

Contig 55496 GCATATGTTGGTCAATGCAGCAAAAGCAAGATCCGTTTGCAGCCTCCCCCATGAAAAATG 55437

INO Gb 961 GCGATGGGCTGGATGCATGCCTGCCTTCTCTGGATGACGAAGAAGAAAAGATTCCTGTGA 1020

||||||||||||||||||||||||||||||||||||||||||||||||||||||||||||

Contig 55436 GCGATGGGCTGGATGCATGCCTGCCTTCTCTGGATGACGAAGAAGAAAAGATTCCTGTGA 55377

INO Gb 1021 CCCCAACAGTCAATAAACgtgagctttctactcaaggactagaacttttacctgattgtt 1080

||||||||||||||||||||||||||||||||||||||||||||||||||||||||||||

Contig 55376 CCCCAACAGTCAATAAACGTGAGCTTTCTACTCAAGGACTAGAACTTTTACCTGATTGTT 55317

INO Gb 1081 atatttcacatcgaaaaatgatttggtttcatctctcacagCTCCAGAGAAGCGCCAACG 1140

||||||||||||||||||||||||||||||||||||||||||||||||||||||||||||

Contig 55316 ATATTTCACATCGAAAAATGATTTGGTTTCATCTCTCACAGCTCCAGAGAAGCGCCAACG 55257

INO Gb 1141 TGCTCCATCAGCATATAACCGCTTTATCAAgtaataatcaccacccatcttgttttttct 1200

||||||||||||||||||||||||||||||||||||||||||||||||||||||||||||

Contig 55256 TGCTCCATCAGCATATAACCGCTTTATCAAGTAATAATCACCACCCATCTTGTTTTTTCT 55197

INO Gb 1201 gcttcaatgaaatgtagctatgattatttggtaaaatccagataaattgtcgtgtcaaag 1260

||||||||||||||||||||||||||||||||||||||||||||||||||||||||||||

Contig 55196 GCTTCAATGAAATGTAGCTATGATTATTTGGTAAAATCCAGATAAATTGTCGTGTCAAAG 55137

INO Gb 1261 tttgaagtaaaatcttacacaactgcactgaaatgggaaatgataaaatgagaattttaa 1320

||||||||||||||||||||||||||||||||||||||||||||||||||||||||||||

Contig 55136 TTTGAAGTAAAATCTTACACAACTGCACTGAAATGGGAAATGATAAAATGAGAATTTTAA 55077

INO Gb 1321 cagAGAAGAGATCCAAAGGCTCAAGGCTAAGCAGCCCAACATAACCCACAAGGAGGCCTT 1380

||||||||||||||||||||||||||||||||||||||||||||||||||||||||||||

Contig 55076 CAGAGAAGAGATCCAAAGGCTCAAGGCTAAGCAGCCCAACATAACCCACAAGGAGGCCTT 55017

INO Gb 1381 CAGCACAGCTGCTAAAAATgtaagtttttcttttatgagtgctgattgcggtcgcttccc 1440

||||||||||||||||||||||||||||||||||||||||||||||||||||||||||||

Contig 55016 CAGCACAGCTGCTAAAAATGTAAGTTTTTCTTTTATGAGTGCTGATTGCGGTCGCTTCCC 54957

INO Gb 1441 caacgg----------------------------------------tggaccacaaacac 1460

|||||| ||||||||||||||

Contig 54956 CAACGGATGCATGACGGAAGATGCTCATCATCTAACTCTTACTTACTGGACCACAAACAC 54897

INO Gb 1461 gacaaatataaaagatggttggtgcaacttgattatcagtgcagatgttttgcagggttg 1520

||||||||||||||||||||||||||||||||||||||||||||||||||||||||||||

Contig 54896 GACAAATATAAAAGATGGTTGGTGCAACTTGATTATCAGTGCAGATGTTTTGCAGGGTTG 54837

INO Gb 1521 ctcacctaacaatcaggtcggcataattcttggaacatgtcatcgtagggaatggcccga 1580

||||||||||||||||||||||||||||||||||||||||||||||||||||||||||||

Contig 54836 CTCACCTAACAATCAGGTCGGCATAATTCTTGGAACATGTCATCGTAGGGAATGGCCCGA 54777

INO Gb 1581 ttaaacagctgtgatattttgtcacgcatttcctcccttttcgcaatgggacatgcagct 1640

||||||||||||||||||||||||||||||||||||||||||||||||||||||||||||

Contig 54776 TTAAACAGCTGTGATATTTTGTCACGCATTTCCTCCCTTTTCGCAATGGGACATGCAGCT 54717

INO Gb 1641 ggcattgagtgagattctttgtgaagtctgaattctaaagacagctgaaaataaagcaag 1700

||||||||||||||||||||||||||||||||||||||||||||||||||||||||||||

Contig 54716 GGCATTGAGTGAGATTCTTTGTGAAGTCTGAATTCTAAAGACAGCTGAAAATAAAGCAAG 54657

INO Gb 1701 ttgctgttttggggggaatatacacaggcatttatttttgtttgactgatttttaactga 1760

||||||||||||||||||||||||||||||||||||||||||||||||||||||||||||

Contig 54656 TTGCTGTTTTGGGGGGAATATACACAGGCATTTATTTTTGTTTGACTGATTTTTAACTGA 54597

INO Gb 1761 ttgcagTGGGCCCACTTCCCTCGCATTCAGTACAAAGGAGACAGAGAGAGCTGCAGCGAG 1820

||||||||||||||||||||||||||||||||||||||||||||||||||||||||||||

Contig 54596 TTGCAGTGGGCCCACTTCCCTCGCATTCAGTACAAAGGAGACAGAGAGAGCTGCAGCGAG 54537

INO Gb 1821 GAGAGACTAGGAAAAG-TTACCTGCTAG 1847

|||||||||||||||| || ||||||||

Contig 54536 GAGAGACTAGGAAAAGTTTCCCTGCTAG 54509

**Supplemental Figure S3.** Amplification products for primer pairs LMINO.

LMINO 1/2 (a), LMINO3/4 (b), LMINO5/6 (c), and LMINO7/8 (d) obtained from DNA samples from each of the parents (A) and of the three F_1_ progenies selected for conducting the segregating generations (B), indicated below the 1.2% agarose gels in TBE 1× buffer. M bp: molecular weight markers in base pairs; Bs: Brazilian seedless; M_1_, M_2_, and M_3_: wild-types (fertiles).


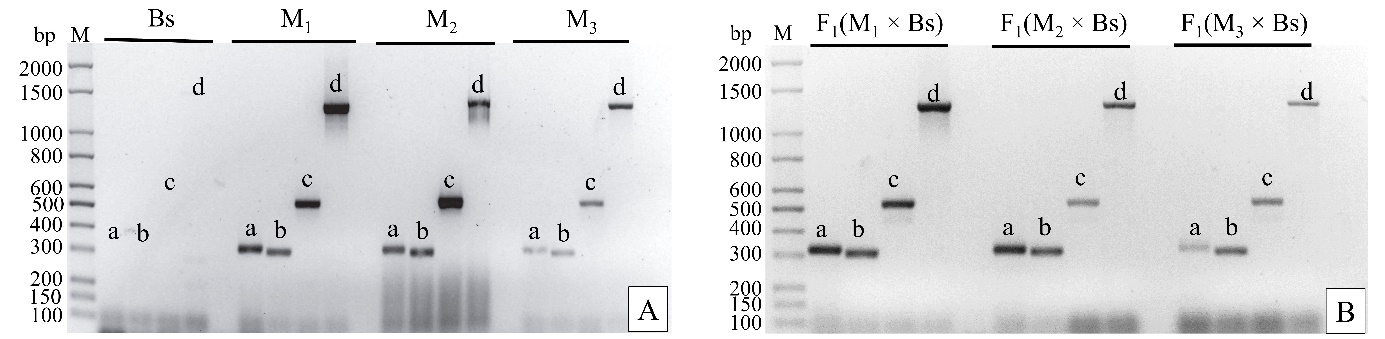

Supplement: Supplementary file 3 — Supplementary file3 (DOCX 478 KB) [file 497_2023_464_MOESM3_ESM.docx]
