# Supplementary material for: Seedless fruit in Annona squamosa L. is monogenic and conferred by INO locus deletion in multiple accessions
Source: Plant Reprod. 2023 May 9;37(2):71–84. doi: 10.1007/s00497-023-00464-9 (PMC11180160; doi:10.1007/s00497-023-00464-9)
Supplement: Supplementary file 4 — Supplementary file4 (DOCX 21 KB) [file 497_2023_464_MOESM4_ESM.docx]

**Seedless fruit in *Annona squamosa* L. is monogenic and conferred by *INO* locus deletion in multiple accessions**

Plant Reproduction

Bruno Rafael Alves Rodrigues¹, Charles S. Gasser^2^, Samy Pimenta¹, Marlon Cristian Toledo Pereira¹ and Silvia Nietsche^3^*

¹ State University of Montes Claros, Department of Agricultural Sciences, CEP 39401-369, Janaúba, MG, Brazil

² Dept. of Molecular and Cellular Biology, University of California, Davis, Davis, CA 95616 USA

^3^ Federal University of Minas Gerais, Institute of Agricultural Science, CEP 39404-547, Montes Claros, Minas Gerais, Brazil

*Author for correspondence: [silvia.nietsche@gmail.com](mailto:silvia.nietsche@gmail.com)

**Supplemental Methods**

Genetic distance estimation

In our segregating populations, no recombination events were detected between the *ino* deletion and the *Brazilian seedless* (*Bs*) phenotypic marker providing a clear indication of linkage on a single chromosome. To estimate the maximum genetic distance between these loci (which we believe to actually be a single locus) we used the following logic and calculations.

1. If the seedless mutation (Bs) and *INO* gene deletion (*ino*) were separate mutations, any cross to a heterozygous line that harbored both a wild-type chromosome and the mutant chromosome harboring these two mutations would allow for the possibility of recombination between the two loci. Such a recombination could be detected if the *ino* and *Bs* traits were shown to be separate in a subsequent generation.
2. Recombination would be observable in a progeny plant that was homozygous for the *ino* deletion where recombination between this locus and *Bs* would lead to the presence of the dominant wild-type locus and produce wild-type ovule/seed phenotype despite the homozygous mutant molecular trait. The absence of seeds in such plants, or the presence of mutant ovules would confirm absence of recombination in either of the two chromosomes.
3. For heterozygous *INO/ino* progeny plants a recombination in the mutant chromosome would not be detected as it would not alter the wild-type phenotype. In contrast, recombination on the wild-type *INO* chromosome would introduce the *Bs* seedless mutant allele and so produce a seedless plant despite the presence of the *INO* wild-type gene. So heterozygous plants allow confirmation of the absence of recombination on the wild-type chromosome.
4. In F2 progeny, both chromosomes derive from plants that were heterozygous in the prior generation (the F1 generation). As a result, in F2 progeny recombination can be detected in one chromosome of heterozygous plants and in two chromosomes of homozygous mutant plants.
5. In BC Bs plants one chromosome derives from a plant that was heterozygous and that could be subject to meiotic recombination (the F1 parent in the backcross). The other chromosome (an *ino/ino Bs/Bs* chromosome) would be from a homozygous plant (the homozygous mutant backcross parent ) and so would not be subject to recombination between these loci. So for BC Bs heterozygous progeny, one chromosome (the *INO* wild type chromosome) could be evaluated by comparing the molecular and phenotypic data. For this generation only one chromosome of homozygous mutant plants could be evaluated (the chromosome deriving from the F1 parent), because the other chromosome, deriving from the *Bs* parent, could not have undergone recombination.
6. Using these criteria, we observed no recombination and could confirm an absence of recombination between *ino* and *Bs* on 114 chromosomes (Supplemental Data File 1).

To delimit the maximum possible distance between Bs and *ino* we used a χ^2^ test. Assuming that *ino* and *Bs* are two separate loci, we can predict the number of expected recombination events based on the genetic distance between the two loci. For example, if we hypothesize a distance of 3.5 centiMorgans (cM), we would predict 3.5% recombination, which for 114 chromosomes would produce a ratio of 110.01 : 3.99 of non-recombinant to recombinant chromosomes. The observed ratio was 114:0. Performing a two-tailed χ^2^ test with one degree of freedom results in a value of 4.35 and a probability of 0.042 that the 3.5% recombination frequency is correct. Thus, the hypothesis that the distance between the “two” loci is 3.5 cM can be rejected due to its having a less than 5% probability. Any larger distance hypothesized produces an even lower probability. Therefore, our results allow conclusion that the distance between *ino* and *Bs* is less than 3.5 cM. Double recombination could potentially mask recombination between the loci, but given a 3.5 cM distance the probability of double recombination is 0.0012, and this negligible probably can be ignored.
